# Cytogenetic Characteristics of Chronic Lymphocytic Leukemia Patients: A Single-Center Study From Morocco

**DOI:** 10.7759/cureus.112825

**Published:** 2026-07-16

**Authors:** Monsif Fadi, Yasmine El Ouafa, Chaimaa Hilali, Fatima Azzahra Lahlou, Nouama Bouanani

**Affiliations:** 1 Oncopathology, Center for Doctoral Studies (CeDoc), Casablanca, MAR; 2 Oncopathology, Cancer Biology and Environment Laboratory, Mohammed VI Faculty of Medicine, Mohammed VI University of Health Sciences (UM6SS), Casablanca, MAR; 3 Interdisciplinary Laboratory of Biotechnology and Health, Mohammed VI University of Health Sciences (UM6SS), Casablanca, MAR; 4 Biochemistry, Health and Environment Laboratory, Ain Chock Faculty of Sciences, Hassan II University, Casablanca, MAR; 5 Hematology, Mohammed VI Faculty of Medicine, Mohammed VI University of Health Sciences (UM6SS), Casablanca, MAR

**Keywords:** chronic lymphocytic leukemia (cll), complex karyotype, fluorescence in situ hybridization (fish), molecular cytogenetics, tumor suppressor gene (tp53) mutation

## Abstract

Introduction and objective: Chronic lymphocytic leukemia (CLL) is the most common adult leukemia, characterized by significant clinical and biological heterogeneity. Cytogenetic abnormalities play a crucial prognostic role and influence treatment decisions. This study aimed to characterize the cytogenetic profile of CLL patients in a Moroccan tertiary hospital and to describe the distribution of major chromosomal abnormalities.

Materials and methods: We conducted a retrospective descriptive study including patients diagnosed with CLL between January 2023 and December 2025 in the Hematology Department. Clinical, demographic, and cytogenetic data were collected from medical records. Cytogenetic analysis was performed using conventional karyotyping and/or fluorescence in situ hybridization (FISH), according to routine availability in daily practice.

Results: A total of 53 patients were included, with a slight male predominance, comprising 28 (52.8%) men and 25 (47.2%) women, resulting in a male-to-female ratio of 1.12. The median age was 57 years, and the mean age was 56.2 years. Conventional karyotyping was performed in 20 of 53 patients (38%). Among the patients who underwent conventional karyotyping, 16 (80%) had a complex karyotype and four (20%) had a normal karyotype, corresponding to 30.2% and 7.5% of the overall cohort, respectively. FISH analysis was available for 46 (86.8%) patients and detected cytogenetic abnormalities in 24 (45.3%) patients. The most frequent abnormality was del(17p)/TP53, identified in 17 (47.2%) patients, followed by del(11q) in nine (25%) patients, del(13q14) in six (16.7%) patients, trisomy 12 in three (8.3%) patients, and del(14q) in one (2.8%) patient. Only one (2.8%) patient had an isolated 13q deletion, which is generally associated with a favorable prognosis.

Conclusion: Cytogenetic abnormalities were frequent in this cohort, mainly TP53/17p deletion and complex karyotype. These results support routine cytogenetic assessment for accurate risk stratification and treatment planning in CLL.

## Introduction

Chronic lymphocytic leukemia (CLL) represents the most frequent hematological malignancy in adults in Western countries. It is defined by a progressive accumulation of monoclonal, mature B lymphocytes in the peripheral blood, bone marrow, lymph nodes, and spleen. Clinically, CLL is characterized by a highly heterogeneous evolution, where some patients remain stable and asymptomatic for many years, whereas others exhibit an aggressive course requiring early therapeutic management [1].

In recent decades, the identification of biological and genetic markers has considerably enhanced the understanding of this clinical variability. Among these parameters, cytogenetic abnormalities hold a central position due to their strong prognostic significance and their influence on treatment decisions. Current clinical guidelines therefore recommend routine cytogenetic assessment at the time of CLL diagnosis [2].

The advent of fluorescence in situ hybridization (FISH) has markedly improved the detection of chromosomal abnormalities, identifying genetic alterations in more than 80% of CLL cases, whereas conventional karyotyping was historically limited by the low proliferative activity of leukemic cells. The most common abnormalities include deletion 13q [del(13q)], trisomy 12, deletion 11q [del(11q)], and deletion 17p [del(17p)], the latter frequently associated with disruption of the TP53 gene [1,2].

Among these lesions, del(13q) is the most frequently observed and is generally associated with a favorable prognosis when isolated. In contrast, del(17p) is considered a high-risk abnormality, associated with aggressive disease evolution, resistance to standard therapies, and reduced overall survival [1]. Patients with del(11q) often present with bulky lymphadenopathy and a more aggressive clinical course, while trisomy 12 is generally classified as an intermediate-risk cytogenetic abnormality [3].

More recently, the concept of complex karyotype, defined by the presence of at least three chromosomal abnormalities, has emerged as an independent adverse prognostic factor associated with poor outcomes and reduced survival, even in the era of targeted therapies [4].

Despite significant progress in the genetic characterization of CLL, data concerning the cytogenetic landscape in Moroccan patients remain limited. A better description of these abnormalities in our local population is essential to improve disease understanding and to allow meaningful comparison with international cohorts.

The objective of this study was to evaluate the cytogenetic profile of patients with CLL followed in a Moroccan center between January 2023 and December 2025 and to analyze the distribution of the main chromosomal abnormalities observed in this cohort.

## Materials and methods

Study design and setting

This was a retrospective, descriptive study evaluating the cytogenetic profile of patients diagnosed with CLL. The study was conducted over a three-year period, from January 2023 to December 2025, in the Hematology Department of Cheikh Khalifa University Hospital, Casablanca, Morocco.

Study population

The study included patients diagnosed with CLL who had complete clinical and biological data available. The diagnosis was established based on clinical, morphological, and immunophenotypic criteria according to internationally accepted standards.

Patients were eligible if they had a confirmed diagnosis of CLL based on immunophenotyping, a Matutes score of 4 or 5, and were followed in the department during the study period. The Matutes score was established using the expression of CD5, CD23, FMC7, surface immunoglobulin (sIg), and CD22/CD79b, with one point assigned for each marker showing the typical immunophenotypic profile of CLL, resulting in a score ranging from 0 to 5. A score of 4 or 5 was considered consistent with typical CLL, whereas scores below 4 were suggestive of an atypical phenotype or another B-cell lymphoproliferative disorder. Patients with a Matutes score below 4, those diagnosed with other B-cell lymphoproliferative disorders, or those with incomplete medical records were excluded.

The Matutes immunophenotypic scoring system and FISH analysis for TP53 deletion are internationally recognized and validated tools that are routinely employed in both clinical practice and research settings, without requiring any specific licensing [5].

Data collection

Data were retrospectively collected from medical records using a standardized data extraction form. The variables analyzed included demographic data (age and sex), clinical and biological characteristics at diagnosis, and cytogenetic findings.

Cytogenetic analysis was performed using conventional karyotyping and/or FISH, depending on availability. The main abnormalities assessed included deletions of 13q, 11q, and 17p, as well as trisomy 12.

FISH analysis was performed using the standard CLL FISH panel routinely available in our institution according to routine clinical practice.

Statistical analysis

Continuous variables were summarized using the mean and median, whereas categorical variables were expressed as frequencies and percentages (N, %). Associations between categorical variables were evaluated using Pearson's chi-square test. A two-sided p-value <0.05 was considered statistically significant. Statistical analyses were performed using IBM SPSS Statistics version 27.0.1 (IBM Corp., Armonk, NY, USA).

## Results

Distribution by sex

We included 53 patients diagnosed with CLL from January 2023 to December 2025. The study population was predominantly male. Among the 53 patients, 28 were men and 25 were women, corresponding to 52.8% and 47.2%, respectively, with a male-to-female sex ratio of 1.12.

Distribution according to age

The median age of the patients was 57 years, while the mean age was 56.2 years. This indicates that the study population was predominantly composed of middle-aged and elderly adults, which is consistent with the epidemiological characteristics of CLL. 

Distribution according to the type of karyotype identified

**Figure 1:**
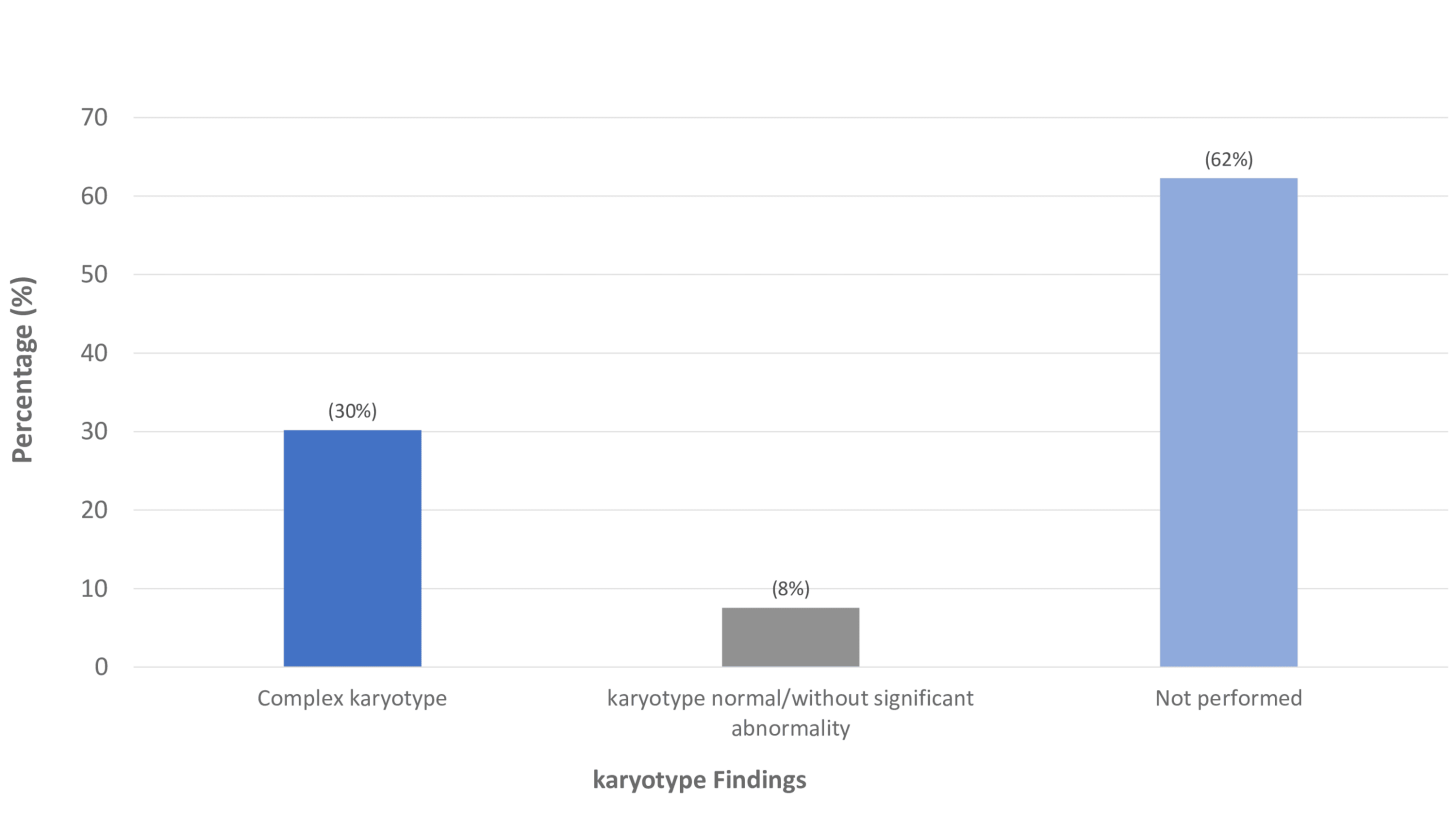
Distribution of conventional karyotype findings (n = 53) Conventional karyotyping was performed in 20 of 53 patients (38%). Among those who underwent karyotyping, 16 (80%) had a complex karyotype and four (20%) had a normal karyotype, corresponding to 30.2% and 7.5% of the overall cohort, respectively. Karyotyping was not performed in 33 patients (62.3%). Data are presented as N (%).

Conventional karyotyping was performed in 20 of the 53 patients (38%), while 33 (62.3%) did not undergo karyotyping. Among the patients who underwent conventional karyotyping, 16 (80%) had a complex karyotype, defined as the presence of three or more chromosomal abnormalities according to Baliakas et al. [4], whereas four (20%) had a normal karyotype without significant chromosomal abnormalities. These findings correspond to 30.2% and 7.5% of the overall study population, respectively (Figure 1).

Different cytogenetic abnormalities observed in our series

**Figure 2:**
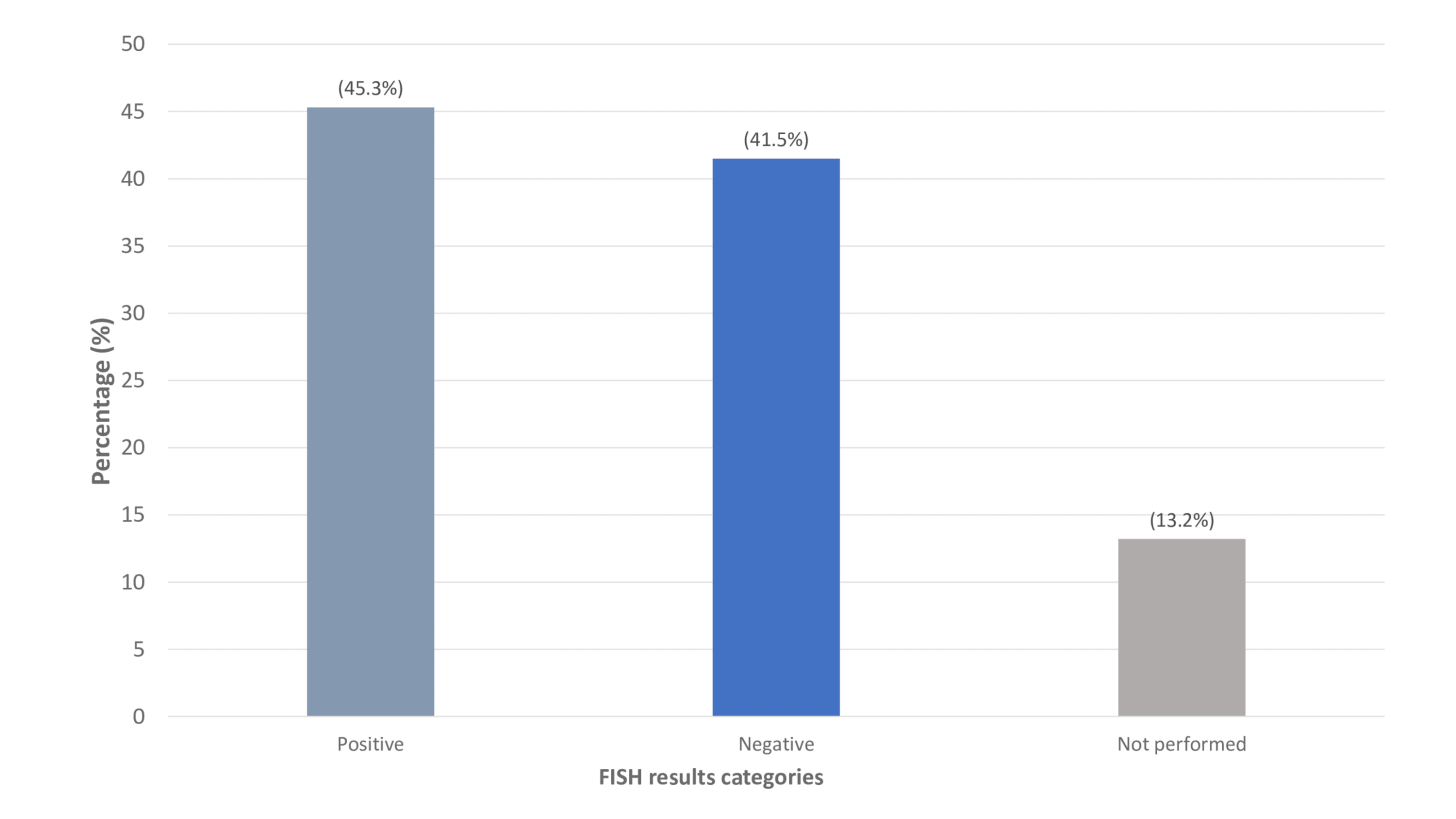
Distribution of FISH results (n = 53) Fluorescence in situ hybridization (FISH) analysis detected at least one cytogenetic abnormality in 24 (45.3%) patients, whereas no detectable abnormality was identified in 22 (41.5%) patients. FISH analysis was not performed in seven (13.2%) patients. Data are presented as N (%).

FISH was carried out in 46 of the 53 patients. Cytogenetic abnormalities were detected in 24 (45.3%) patients, while 22 (41.5%) patients showed no detectable abnormality. FISH analysis was not performed in the remaining seven (13.2%) patients (Figure 2). 

**Figure 3:**
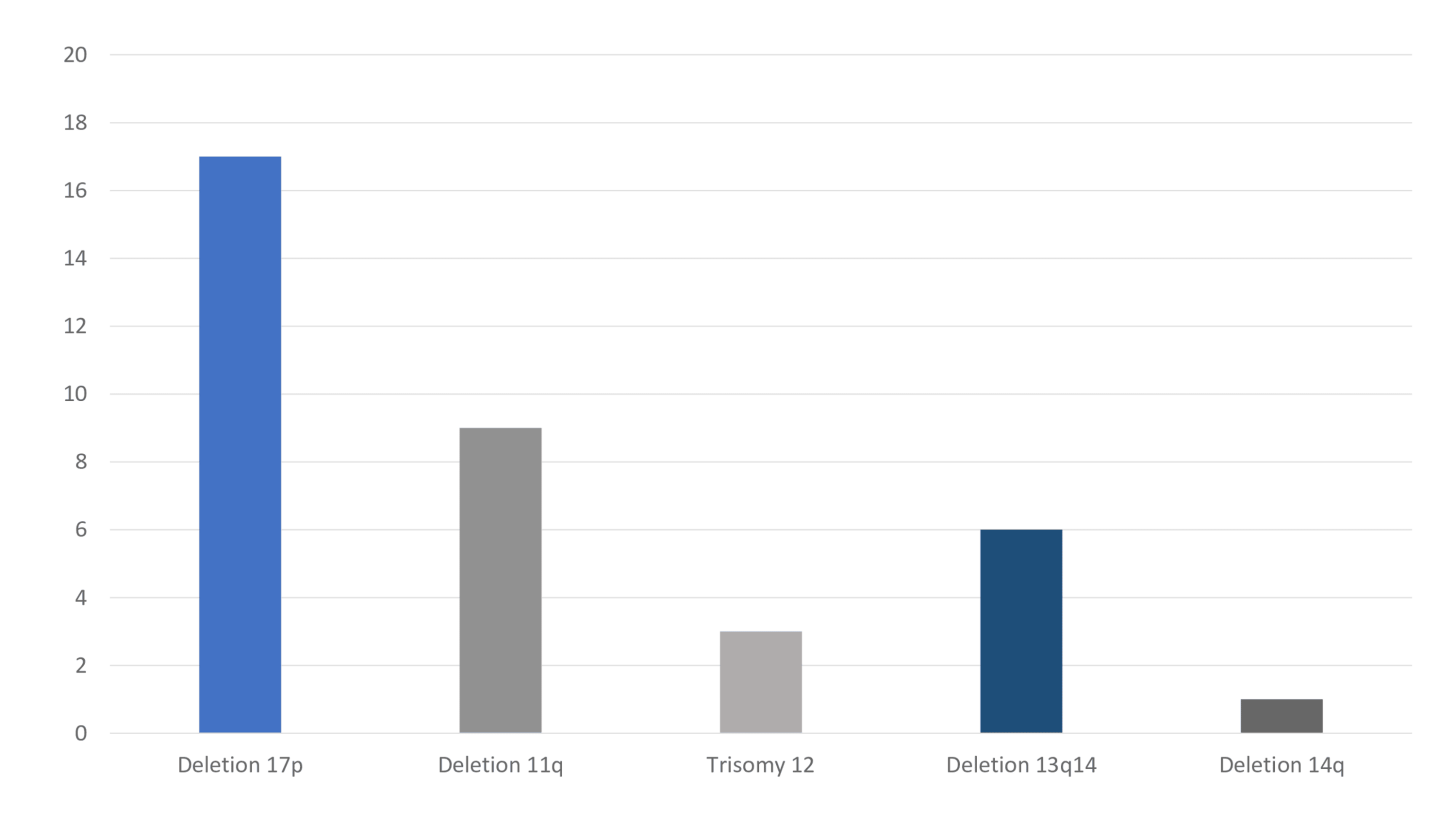
Distribution of the 36 cytogenetic abnormalities detected in 24 FISH-positive patients (n = 53) A total of 36 cytogenetic abnormalities were identified among 24 patients with at least one fluorescence in situ hybridization (FISH) abnormality, indicating that some patients harbored more than one cytogenetic lesion. Percentages were calculated based on the total number of detected cytogenetic abnormalities (n = 36).

A total of 36 cytogenetic abnormalities were identified among 24 patients with at least one FISH abnormality. Deletion 17p involving the TP53 locus was the most frequent abnormality, accounting for 17 of the 36 detected abnormalities (47.2%), followed by deletion 11q (9/36, 25%), deletion 13q14 (6/36, 16.7%), trisomy 12 (3/36, 8.3%), and deletion 14q (1/36, 2.8%) (Figure 3).

## Discussion

CLL is the most common leukemia in adults in Western countries, accounting for approximately 25-30% of all leukemias [6,7,8]. It is characterized by the clonal accumulation of mature B lymphocytes in the peripheral blood, bone marrow, and lymphoid tissues [7]. Clinically, CLL exhibits a markedly heterogeneous course, ranging from indolent disease requiring no immediate therapy to rapidly progressive forms necessitating early treatment [8,9]. This variability is largely driven by underlying genetic and cytogenetic abnormalities, which have become essential for risk stratification and therapeutic decision-making in contemporary CLL management [10,11].

In the present study, 53 patients diagnosed with CLL were analyzed, with a mean age of 56.2 years and a median age of 57 years. These values are slightly lower than those reported in Western populations, where the median age at diagnosis is approximately 65 years [7,8]. This finding may suggest a relatively younger age at diagnosis in Moroccan patients, a pattern also suggested in other North African series, although larger epidemiological studies are required to confirm this trend.

Regarding sex distribution, males represented 52.8% of the cohort, with a male-to-female ratio of 1.12. This slight male predominance is consistent with the well-established epidemiological pattern of CLL, which is generally more frequent in males across most populations [7,8].

From a cytogenetic perspective, FISH analysis was performed in 46 of the 53 patients. Deletion 17p involving the TP53 locus was the most frequently detected abnormality, identified in 17 patients (47.2% of FISH-positive cases). This prevalence is considerably higher than that reported in newly diagnosed CLL cohorts, where del(17p) is typically observed in 5-10% of patients [9,10]. This discrepancy is most likely explained by referral bias, as tertiary care centers often receive more advanced or high-risk cases. The retrospective design of the study may also have contributed to this enrichment.

TP53 disruption remains one of the most powerful adverse prognostic markers in CLL, being strongly associated with resistance to chemoimmunotherapy, rapid disease progression, and reduced overall survival [10,11]. The high frequency observed in our cohort suggests a population enriched in high-risk genetic features, with important therapeutic implications, particularly the preferential use of targeted agents, such as Bruton tyrosine kinase (BTK) inhibitors and BCL-2 inhibitors, which are now recommended as first-line therapy in this setting [11].

Deletion 11q was identified in nine (25%) patients, trisomy 12 in three (8.3%) patients, and deletion 13q14 in six (16.7%) patients, including one case of isolated del(13q), which is classically associated with a favorable prognosis and represents the most frequent cytogenetic abnormality in unselected CLL populations [9,10]. Deletion 14q was observed in one (2.8%) patient.

Conventional karyotyping was performed in 20 of the 53 patients (38%). Among those who underwent conventional karyotyping, 16 (80%) had a complex karyotype, whereas four (20%) had a normal karyotype. Because conventional karyotyping was not performed systematically and was more likely to be requested in patients with suspected high-risk disease, this high proportion of complex karyotypes among tested patients may reflect selection bias and should not be interpreted as the prevalence in the overall cohort. Complex karyotype is increasingly recognized as an independent adverse prognostic factor in CLL, particularly when defined as the presence of three or more chromosomal abnormalities [4,12].

The low proportion of normal karyotype further highlights the significant cytogenetic burden observed in this cohort. The complementary use of conventional karyotyping and FISH allowed a more comprehensive characterization of chromosomal abnormalities, in accordance with current international recommendations [11].

From a therapeutic perspective, the high prevalence of del(17p) and complex karyotype has direct clinical implications. Patients harboring these abnormalities are unlikely to benefit from conventional chemoimmunotherapy and should preferentially receive targeted therapies. BTK inhibitors, such as ibrutinib and acalabrutinib, as well as the BCL-2 inhibitor venetoclax, have demonstrated substantial efficacy in this high-risk population and are currently recommended as frontline treatment options in international guidelines [11,13].

The therapeutic landscape of CLL has evolved considerably with the introduction of targeted therapies, which have largely replaced chemoimmunotherapy in many clinical settings. Covalent BTK inhibitors, including ibrutinib and acalabrutinib, provide sustained disease control through continuous inhibition of B-cell receptor signaling, whereas venetoclax-based regimens offer the advantage of fixed-duration treatment and can achieve deep responses with high rates of undetectable minimal residual disease [14,15]. The selection of treatment should be individualized according to patient age, comorbidities, genetic profile, treatment objectives, and drug availability. In patients with TP53 abnormalities or complex karyotype, both BTK inhibitors and venetoclax-based therapies have consistently demonstrated superior clinical outcomes compared with conventional chemoimmunotherapy and are therefore recommended as the preferred treatment options [11,14,15]. More recently, combinations and sequential strategies involving BTK and BCL-2 inhibitors have shown promising efficacy, with the potential to further improve the depth and durability of response while reducing the risk of disease progression [15,16]. These advances reinforce the importance of comprehensive cytogenetic and molecular evaluation at diagnosis to optimize therapeutic decision-making and support a personalized approach to CLL management [11,16].

Limitations of the study

Several limitations should be acknowledged. First, the retrospective single-center design introduces selection bias, as the study was conducted in a tertiary referral center that tends to concentrate patients with more advanced or high-risk disease, which may explain the unusually high prevalence of del(17p). Second, the relatively small sample size (n = 53) limits statistical power and generalizability. Third, conventional karyotyping was not systematically performed in all patients, which may have led to an underestimation of complex karyotype frequency. Conventional karyotyping was not performed in 62 % of patients in our series. This can be explained by the limitations encountered in a resource-constrained tertiary care setting, where the specialized culture techniques required for CLL cytogenetic analysis, particularly the use of DSP30 and interleukin-2 stimulation, are not routinely available. In addition, given the retrospective nature of the study, karyotyping was not systematically requested at diagnosis, as FISH was often used as the primary cytogenetic assessment. These findings highlight the challenges associated with implementing a comprehensive cytogenetic evaluation in routine practice and emphasize the importance of performing a complete cytogenetic workup at diagnosis, in line with current international recommendations. Fourth, the absence of molecular data, including IGHV mutational status and TP53 mutation analysis by sequencing, limits the completeness of the prognostic assessment, as FISH alone does not capture all clinically relevant genomic alterations [9,11]. Finally, the lack of national epidemiological data on CLL in Morocco limits external comparison and interpretation of demographic patterns.

Despite these limitations, this study provides important preliminary data on the cytogenetic landscape of CLL in a Moroccan tertiary care setting. It contributes to the growing understanding of CLL in North Africa. Larger prospective multicenter studies incorporating comprehensive genomic profiling are needed to validate these findings and to better define the molecular epidemiology of CLL in this population.

## Conclusions

This study provides an overview of the cytogenetic profile of Moroccan patients with CLL treated at our institution. A high prevalence of adverse cytogenetic abnormalities, particularly TP53/17p deletion and complex karyotype, was observed, suggesting a population enriched in high-risk genetic features. These findings highlight the major prognostic value of cytogenetic assessment in the initial evaluation of CLL patients and its importance in guiding therapeutic decision-making. Although limited by its retrospective single-center design and relatively small sample size, this study contributes to the characterization of CLL in Morocco and provides valuable data for comparison with international cohorts. Larger multicenter studies integrating cytogenetic and molecular markers are warranted to further improve the understanding of the biological and prognostic landscape of CLL in the Moroccan population.
